# Author Correction: Power laws in species’ biotic interaction networks can be inferred from co-occurrence data

**DOI:** 10.1038/s41559-024-02508-3

**Published:** 2024-07-22

**Authors:** Nuria Galiana, Jean-François Arnoldi, Frederico Mestre, Alejandro Rozenfeld, Miguel B. Araújo

**Affiliations:** 1 Department of Biogeography and Global Change, National Museum of Natural Sciences, Madrid, Spain; 2 Centre National de la Recherche Scientifique, Experimental and Theoretical Ecology Station, Moulis, France; 3 Rui Nabeiro Biodiversity Chair, Mediterranean Institute for Agriculture, Environment and Development, University of Évora, Évora, Portugal; 4 INTELYMEC Group, Centro de Investigaciones en Física e Ingeniería del Centro Centro de Investigaciones en Física e Ingeniería del Centro de la Provincia de Buenos Aires – Universidad Nacional del Centro de la Provincia de Buenos Aires – Consejo Nacional de Investigaciones Científicas y Técnicas, Olavarría, Argentina

**Keywords:** Biogeography, Ecological networks

Correction to: *Nature Ecology & Evolution* 10.1038/s41559-023-02254-y, published online 27 November 2023.

This paper was originally published under standard Springer Nature license (© The Author(s), under exclusive licence to Springer Nature Limited). It is now available as an open-access paper under a Creative Commons Attribution 4.0 International license, © The Author(s). The error has been corrected in the HTML and PDF versions of the article.

