# Supplementary material for: Power laws in species’ biotic interaction networks can be inferred from co-occurrence data
Source: Nat Ecol Evol. 2023 Nov 27;8(2):209–17. doi: 10.1038/s41559-023-02254-y (PMC11263125; doi:10.1038/s41559-023-02254-y)
Supplement: Supplementary file 1 — Supplementary Text 1. Dataset description. Table 1. Basic network properties of each dataset. Text 2. Mathematical transformations of degree distributions: how to get from an exponential to a power law. Table 2. Values of the per-site interaction rate (P) for each dataset. Table 3. Degree distribution fits for each dataset. [file 41559_2023_2254_MOESM1_ESM.pdf]

nature ecology & evolution

Article https://doi.org/10.1038/s41559-023-02254-y

Power laws in species' biotic interaction networks can be inferred from co-occurrence data

In the format provided by the authors and unedited

SUPPLEMENTARY MATERIAL

List of content:

- Supplementary Text 1. Dataset description.

- Supplementary Table 1. Basic network properties of each dataset.

- **Supplementary Text 2.** Mathematical transformations of degree distributions: how to get from an exponential to a power law.

- **Supplementary Table 2.** Values of the per-site interaction rate (p) for each dataset.

- **Supplementary Table 3.** Degree distribution fits for each dataset.

Supplementary Text 1. Dataset description.

(1) GARRAF PP: Plant-pollinator interaction networks in Mediterranean scrubland in the Garraf Natural Park

- **Study area:** Garraf Natural Park, Catalunya, Spain
- **Interaction types:** Plant-pollinator interactions.
- **Type of system:** Mediterranean scrubland.
- **Number and extent of sampling units:** 40 local patches of the same size (40x30 m) within a homogeneous landscape connected through dispersal. Total area: 40 km^2. Distance between patches: 520 to 1440 m.
- **Taxonomic resolution of the nodes:** Species level.
- **Sampling procedure (of species and interactions):** 1) Counted number of visits per open flower by each pollinator species. 2) Sampled pollinator and plant species.
- **Publication reference:** Reverté S., Bosch J., Arnan X., Roslin T., Stefanescu C., Calleja J. A., Molowny-Horas R., Hernández-Castellano C., Rodrigo A. 2019. Spatial variability in a plant-pollinator community across a continuous habitat: high heterogeneity in the face of apparent uniformity. *Ecography* 42: 1–11, 2019. doi: 10.1111/ecog.04498

(2) GARRAF PP2: Plant-pollinator interaction networks in Mediterranean scrubland in the Garraf Natural Park

- **Study area:** Garraf Natural Park, Catalunya, Spain
- **Interaction types:** Plant-pollinator interactions.
- **Type of system:** Mediterranean scrubland.
- **Number and extent of sampling units:** 21 local patches of the same size (40x40 m) within a homogeneous landscape connected through dispersal. Total area: 32 km^2. Distance between patches: 585 to 1345 m.
- **Taxonomic resolution of the nodes:** Species level.
- **Sampling procedure (of species and interactions):** 1) Counted number of visits per open flower by each pollinator species. 2) Sampled pollinator and plant species in the area.
- **Publication reference:** Reverté S., Bosch J., Arnan X., Roslin T., Stefanescu C., Calleja J. A., Molowny-Horas R., Hernández-Castellano C., Rodrigo A. 2019. Spatial variability in a plant-pollinator community across a continuous habitat: high heterogeneity in the face of apparent uniformity. *Ecography* 42: 1–11, 2019. doi: 10.1111/ecog.04498

(3) MONTSENY: Plant-pollinator interaction networks within dense forest in Montseny Natural Park, Catalunya, Spain.

- **Study area:** Montseny Natural Park, Catalunya, Spain
- **Interaction types:** Plant-pollinator interactions
- **Type of system:** Local patches (clearings) within a dense forest matrix
- **Number and extent of sampling units:** 18 local patches of the same size (25x25 m). Total area: 18.7 km^2. Distance between patches 550 to 2050 m.
- **Taxonomic resolution of the nodes:** Species level.

- **Sampling procedure (of species and interactions):** 1) Counted number of visits per open flower by each pollinator species. 2) Sampled pollinator and plant species in the area.
- **Publication reference:** C. Hernández-Castellano, A. Rodrigo, J. M. Gómez, C. Stefanescu, J. A. Calleja, S. Reverté, J. Bosch. 2020. A new native plant in the neighborhood: effects on plant-pollinator networks, pollination and plant reproductive success. *Ecology* doi.org/10.1002/ecy.3046

(4) NAHUEL: Plant-pollinator networks in Nahuel Huapi National Park, Argentina.

- **Study area:** Nahuel Huapi National Park and surrounding areas in Neuquén and Río Negro provinces, Argentina
- **Interaction types:** Plant-pollinator interactions (flower visits).
- **Type of system:** Eight sites with native temperate forest, four grazed with domestic cattle and four ungrazed, located in an area of ca. 20x50 km.
- **Number and extent of sampling units:** Eight sites of 6-12 ha.
- **Taxonomic resolution of the nodes:** Species level
- **Sampling procedure (of species and interactions):** Data were collected throughout one flowering season, with weekly sampling of each site (a pair of sites per day), with multiple 5 min observation periods per site and date. Interactions were determined by observed visits to flowers. Links are quantified as the total number of visits recorded in the study.
- **Publication reference:** Vázquez, D. P. & Simberloff, D. 2003. Changes in interaction biodiversity induced by an introduced ungulate Ecology Letters, 6, 1077-1083

(5) GOTTIN PP: Plant-pollinator interaction networks on fragmented calcareous grasslands of Germany.

- **Study area:** Göttingen, central Germany
- **Interaction types:** Plant-pollinator interactions
- **Type of system:** Calcareous grassland. Semi-natural habitat of high conservation value due to their high biodiversity (plants and insects in particular). These grasslands are heavily fragmented due to agricultural landscape simplification and intensification.
- **Number and extent of sampling units:** 32 fragments. Area size of fragments ranged from 314– 51,395 m^2.
- **Taxonomic resolution of the nodes:** Species level.
- **Sampling procedure (of species and interactions):** Flower visitors (wild bees and hoverflies; assumed to be pollinators of visited plants) were sampled via four five-minute-transect walks six times from April to September 2004 within a 4 m corridor. Data from the 5-min-transects of all six sampling events were pooled per grassland fragment. Specimens were either identified on the wing or caught with a net and identified in the lab. The plant species visited was recorded for each specimen.
- **Publication reference:** Grass, I., Jauker, B., Steffan-Dewenter, I., Tscharntke, T., & Jauker, F. (2018). Past and potential future effects of habitat fragmentation on structure and stability of plant–pollinator and host–parasitoid networks. *Nature ecology & evolution*, 1.

(6) GOTTIN HP: host-parasitoid interaction networks on fragmented calcareous grasslands of Germany.

- **Study area:** Göttingen, central Germany
- **Interaction types:** Host-parasitoid interactions
- **Type of system:** Calcareous grassland. Semi-natural habitat of high conservation value due to their high biodiversity (plants and insects in particular). These grasslands are heavily fragmented due to agricultural landscape simplification and intensification.
- **Number and extent of sampling units:** 32 fragments. Area size of fragments ranged from 314– 51,395 m^2.
- **Taxonomic resolution of the nodes:** Species level in most cases (some hosts or parasitoids identified to genus level and then assigned to morphospecies).
- **Sampling procedure (of species and interactions):** Parasitoids/parasites and hosts were sampled using trap nests at the same sites. Trap nests consisted of bundles of reed internodes of common reed *Phragmites australis* (about 150–180 reed internodes of 2–10 mm diameter in plastic tubes of 10 cm diameter per trap nest) exposed at a height of 100–120 cm. Depending on the fragment size, 4–6 wooden posts with 2 trap nests each were used: 4 posts (8 trap nests) in 11 small fragments, 5 posts (10 trap nests) in 13 medium fragments, 6 posts (12 trap nests) in eight large fragments. The trap nests were spread regularly over study sites and exposed at the beginning of the flowering period (mid-April) until autumn (beginning October). Afterwards, trap nests were stored in a climate chamber at 4°C and occupied reed internodes were opened. For each nest, the number of brood cells and number of parasitized cells were recorded. We identified host and parasitoid identities to genus or species level as far as possible using larvae and nest characteristics. Because *Osmia rufa* overwinter as adults, these cocoons were opened to check for parasitoids. All other nests were stored separately in test tubes closed with a wad of cotton wool. Tubes were exposed to room temperature (ca. 20°C) to end diapause. Reared adults were identified to species level.
- **Publication reference:** Grass, I., Jauker, B., Steffan-Dewenter, I., Tscharntke, T., & Jauker, F. (2018). Past and potential future effects of habitat fragmentation on structure and stability of plant–pollinator and host–parasitoid networks. *Nature ecology & evolution*, 1.

(7) QUERCUS: Host-specific gallers and leaf-miners in pedunculate oaks in Finland.

- **Study area:** natural communities of specialist insect-herbivores and their natural enemies on the pedunculate oak, *Quercus robur* in the southwest coast of Finland.
- **Interaction types:** Host-parasitoid interactions (host-specific gallers and leaf-miners in pedunculate oaks)
- **Type of system:** Naturally fragmented landscape of oak trees in the archipelago of SW Finland.
- **Number and extent of sampling units:** 22 oak trees.
- **Taxonomic resolution of the nodes:** Species level.
- **Sampling procedure (of species and interactions):** sampling was conducted three times in 2006: in May-June, in late July, and in September in 2006. During each sampling event, a standardized volume of foliage (30 half-meter branches per tree) was collected with the aid of a pole pruner, and all galls and leaf-mines present were recorded. Interactions were quantified by rearing of predators.

- **Publication reference:** Kaartinen, R., & Roslin, T. (2011). Shrinking by numbers: landscape context affects the species composition but not the quantitative structure of local food webs. *Journal of Animal Ecology*, *80*(3), 622-631.

(8) OLOT: Host-parasite interaction networks within a mosaic of forest/agricultural landscape in Olot, Catalunya, Spain.

- **Study area:** Olot, Catalunya, Spain
- **Interaction types:** Host-parasite (Cavity-nesting bee/wasps and their associated parasites) interactions
- **Type of system:** Mosaic landscape of mixed forest and extensive agricultural land
- **Number and extent of sampling units:** 14 local patches. Total area: 100 km^2. Distance between patches 1.4 to 13 km.
- **Taxonomic resolution of the nodes:** Species level.
- **Sampling procedure (of species and interactions):** Nests of bees and wasps collected with trap-nests. Hosts and parasites reared from each nest. Interactions quantified as the number of cells of each host species attacked by each parasite species.
- **Publication reference:** S. Osorio, X. Arnan, E. Bassols, N. Vicens, J. Bosch. 2015. Local and landscape effects in a host–parasitoid interaction network along a forest–cropland gradient. *Ecological Applications* 25: 1869–1879.

(9) GARRAF HP: Host-parasite interaction networks in Mediterranean scrubland in the Garraf Natural Park

- **Study area:** Garraf Natural Park, Catalunya, Spain
- **Interaction types:** Host-parasite (Cavity-nesting bee/wasps and their cleptoparasites, parasitoids and nest predators) interactions.
- **Type of system:** Mediterranean scrubland.
- **Number and extent of sampling units:** 25 local patches. Total area: 33 km^2. Distance between patches: 585 to 1354 m.
- **Taxonomic resolution of the nodes:** Species level.
- **Sampling procedure (of species and interactions):** Nests of bees and wasps collected with trap-nests. Hosts and parasites reared from each nest. Interactions quantified as the number of cells of each host species attacked by each parasite species.
- **Publication reference:** A. Torné-Noguera, X. Arnan, A. Rodrigo, J. Bosch. 2020. Spatial variability of hosts, parasitoids and their interactions across a homogeneous landscape. *Ecology & Evolution* 10: 3696-3705.

(10) GALPAR: Host-parasitoid interactions observed on willow tree species (*Salix* spp.) across Europe.

- **Study area:** Europe - from Italy in the south to Northern Norway
- **Interaction types:** Host-parasitoid interactions
- **Type of system:** Different habitats where species belonging to the Salix genus are found
- **Number and extent of sampling units:** 641 sites. Area size of sites varied between 0.01 and 1 ha depending on the size of individual trees.

- **Taxonomic resolution of the nodes:** Trees and herbivores are resolved to the species level. All trees belong to the Salix genus. All herbivores are galling sawflies. Parasitoids are sometimes resolved to the genus level.
- **Sampling procedure:** Collection and counting of galls produced by the galling sawflies on the trees to identify the herbivore species, and rearing of parasitoids in the laboratory to identify them.
- **Publication reference:** Kopelke, J. P., Nyman, T., Cazelles, K., Gravel, D., Vissault, S., & Roslin, T. (2017). Food-web structure of willow-galling sawflies and their natural enemies across Europe. *Ecology*, *98*(6), 1730-1730.

Supplementary Table 1. Basic network properties of each dataset. Number of species and links in both networks (co-occurrence and realised network of interactions) for each dataset.

Dataset	Sites	Species		Co-occurrence links	Realised links
		Consumers	Resources		
Garraf PP	40	170	24	1355	325
Garraf PP2	21	303	23	3908	967
Montseny	18	194	61	3856	707
Gottin PP	32	138	101	6437	781
Nahuel	8	90	14	938	163
Garraf HP	25	41	26	619	90
Quercus	22	56	23	1192	133
Olot	14	20	29	558	93
Gottin HP	32	21	37	524	108
Galpar	374	126	96	3260	939

Supplementary Text 2. Mathematical transformations of degree distributions: how to get from an exponential to a power law

A network's degree distribution $P(k > x)$ is a positive, decreasing function of $x \geq 0$, defined as the probability that a randomly chosen node has a degree k larger than x. In our paper we compare two kinds of degree distributions computed on empirical networks. The first is for the number d of actual biotic interactions, which we accordingly note $P(d > x)$. The second is the distribution of the number c of co-occurrence links, denoted $P(c > x)$. Because co-occurrence is a condition for interaction in those networks, for any node we have that $d \leq c$.

To understand how a systematic relationship between d and c translates into a predictable relationship between the two degree distributions, suppose that $d = F(c)$, with F a strictly increasing function and such that $F(c) \leq c$ for all realised values of c. We can then write $P(d > x) = P(F(c) > x) = P(c > F^{-1}(x))$. This encodes the formal relationship between the two distributions.

Let us consider a basic example before introducing a family of functions that interpolate between various scenarios (depicted in Figure 3 of the main text). If the number of interactions is proportional to the number of co-occurrences, so if $d = fc$, then $P(d > x) = P(c > \frac{x}{f})$. The two distributions are simply rescalled versions of one another. In particular if $P(c > x) = e^{-Ax}$ (an exponential function, which is what we tend to find in the data analysed) then $P(d > x) = e^{-\frac{A}{f}x}$ which is also an exponential, only steeper (which is *not* what we tend to find in the data).

To consider at once various relationships between d and c, let us define $d = F(c) = \frac{K}{a}(e^{ac} - 1)$ (we specify the constant K below). Its inverse is $c = F^{-1}(d) = \frac{1}{a}\log(1 + \frac{a}{K}d)$. Note that for small a, we have that $F(c) \approx K\left(c + \frac{ac^2}{2} + \cdots\right)$, and $F^{-1}(d) \approx \frac{1}{K}(d - \frac{a}{2K}d^2 + \cdots)$. Thus for $a \approx 0$ we recover the simple proportional relationship from above (and see that we should choose $K = f$ if $a = 0$). For $a > 0$ species with larger numbers of co-occurrence keep a larger proportion of those as actual biotic interactions. For $a < 0$ the opposite is true. Now, if $P(c > x) = e^{-Ax}$ then $P(d > x) = P\big(c > F^{-1}(x)\big) = (1 + \frac{a}{K}x)^{-A/a}$. This function converges to a power law $\sim x^{-A/a}$ for large enough values of a (which tends to be the shape of the empirical data analysed). For $a < 0$ on the other hand, the probability drops to zero at $x = K/|a|$, so a much stepper decline than for the distribution of co-occurrence links.

To be complete we can specify K such that the overall fraction of links kept is constant and equal to f as a varies, so enforcing $E(d) = fE(c)$. For the special case of an exponential distribution of co-occurrence links, so when $P(c > x) = e^{-Ax}$, then $fE(c) = f/A$. On the other hand, $E(d) = \frac{K(E(e^{ac})-1)}{a} = K(E(e^{ac}) - 1)/a$. Given that

$E(e^{ac}) = \int e^{ax} \frac{dP(c<x)}{dx} dx = \int e^{-(A-a)x} A dx = \frac{A}{A-a}$ we get to the simple expression $K = f\left(1 - \frac{a}{A}\right)$.

Supplementary Table 2. Values of the per-site interaction rate (p) for each dataset. We selected the appropriate per-site interaction rates for each dataset in order to prune the co-occurrence network up to the point where we had the same number of links that in the empirical networks of biotic interactions (see methods). The mean value across datasets is $\boldsymbol{p = 0.077 \pm 0.028}$.

Dataset	Per-site interaction rate (p)
Garraf PP	0.1
Garraf PP2	0.096
Montseny	0.099
Gottin PP	0.044
Nahuel	0.065
Garraf HP	0.076
Quercus	0.023
Olot	0.07
Gottin HP	0.074
Galpar	0.12

Supplementary Table 3. The degree distribution of each ecological network was fitted to four different functions that have been identified as typical of the shapes observed in degree distributions in ecological networks: power law, truncated power law, lognormal and exponential. After fitting these functions we selected the most parsimonious one using the Akaike information criterion (AIC). The cumulative probabilities $Pc(k)$, for $\geq k$, where $P(k)$ is the probability a species has k resources in the network. The degree distribution of the co-occurrence networks were generally best fitted by an exponential function ($P(k) \sim e^{-k/\xi}$), while the degree distributions of the realised networks were best fitted by a power-law ($P(k) \sim k^{-\gamma}$) or a truncated power-law ($P(k) \sim k^{-\gamma}e^{-k/\xi}$), where $e^{-k/\xi}$ introduces a cut-off at some characteristic scale (ξ). The table below provides the fit of the parameters of the best function for each dataset and type of network.

Dataset	Network Consumers	Param.	Estimate	Std. Error	t-value	Model
Garraf PP	realised	γ	1.49	0.05	31.65***	Power-law
Garraf PP2	realised	ξ	10.44	0.88	11.83***	Truncated Power-law
		γ	0.18	0.03	5.43***	
Montseny	realised	ξ	10.10	2.81	3.59***	Truncated Power-law
		γ	0.55	0.10	5.45***	
Gottin PP	realised	ξ	26.75	4.33	6.17***	Truncated Power-law
		γ	0.61	0.03	23.14***	
Nahuel	realised	γ	1.54	0.06	23.49***	Power-law
Garraf HP	realised	γ	1.12	0.07	15.41***	Power-law
Quercus	realised	γ	1.15	0.06	19.72***	Power-law
Olot	realised	γ	0.70	0.07	9.22***	Power-law
Gottin HP	realised	γ	0.67	0.02	22.24***	Power-law
Galpar	realised	ξ	48.32	5.41	8.93***	Truncated Power-law
		γ	0.60	0.01	49.98***	

Dataset	Network Resources	Param.	Estimate	Std. Error	t-value	Model
Garraf PP	realised	ξ	118.23	29.52	4.00**	Truncated Power-law
		γ	0.45	0.01	32.65***	
Garraf PP2	realised	ξ	25.91	0.51	50.58***	Exponential
Montseny	realised	ξ	33.62	5.61	5.99***	Truncated Power-law
		γ	0.29	0.03	10.62***	
Gottin PP	realised	ξ	13.92	1.54	9.03***	Truncated Power-law
		γ	0.25	0.03	7.24***	
Nahuel	realised	ξ	12.21	1.04	11.74***	Exponential
Garraf HP	realised	γ	0.88	0.05	15.72***	Power-law
Quercus	realised	ξ	6.96	0.62	11.20***	Exponential
Olot	realised	γ	0.71	0.07	9.22***	Power-law
Gottin HP	realised	γ	0.89	0.10	8.41***	Power-law
Galpar	realised	ξ	5.57	0.27	-11.73***	Truncated Power-law
		γ	-0.42	0.036	19.95***	

Dataset	Network consumers	Param.	Estimate	Std. Error	t-value	Model
Garraf PP	co-occurrence	ξ	2.81	0.10	27.4***	Truncated Power-law
		γ	-0.98	0.04	-21.74***	
Garraf PP2	co-occurrence	ξ	38.17	1.73	22.05***	Exponential
Montseny	co-occurrence	ξ	22.55	1.04	21.52***	Exponential
Gottin PP	co-occurrence	ξ	55.02	2.05	26.73***	Exponential
Nahuel	co-occurrence	ξ	2.75	0.29	9.23***	Truncated Power-law
		γ	-1.34	0.16	-8.35***	
Garraf HP	co-occurrence	ξ	20.28	2.18	9.30***	Exponential
Quercus	co-occurrence	ξ	31.86	6.17	5.16***	Exponential
Olot	co-occurrence	ξ	47.41	6.57	7.21***	Exponential
Gottin HP	co-occurrence	ξ	33.03	3.56	9.26***	Exponential
Galpar	co-occurrence	ξ	27.21	1.24	21.90***	Truncated Power-law
		γ	0.02	0.01	1.67*	

Dataset	Network Resources	Param.	Estimate	Std. Error	t-value	Model
Garraf PP	co-occurrence	ξ	64.68	3.07	21.07***	Exponential
Garraf PP2	co-occurrence	γ	0.18	0.02	8.24***	Power-law
Montseny	co-occurrence	ξ	88.35	5.16	17.10***	Exponential
Gottin PP	co-occurrence	ξ	75.60	2.83	26.64***	Exponential
Nahuel	co-occurrence	ξ	112.52	17.09	6.58***	Exponential
Garraf HP	co-occurrence	ξ	14.69	0.66	22.06***	Exponential
Quercus	co-occurrence	ξ	73.32	7.42	9.87***	Exponential
Olot	co-occurrence	ξ	24.45	5.68	4.30***	Exponential
Gottin HP	co-occurrence	ξ	19.44	2.68	7.23***	Exponential
Galpar	co-occurrence	ξ	43.73	2.93	14.90***	Exponential
