# Supplementary material for: Power laws in species’ biotic interaction networks can be inferred from co-occurrence data
Source: Nat Ecol Evol. 2023 Nov 27;8(2):209–17. doi: 10.1038/s41559-023-02254-y (PMC11263125; doi:10.1038/s41559-023-02254-y)
Supplement: Supplementary file 2 — Reporting Summary [file 41559_2023_2254_MOESM2_ESM.pdf]

nature portfolio

Corresponding author(s): Dr. Nuria Galiana

Last updated by author(s): Oct 3, 2023

Reporting Summary

Nature Portfolio wishes to improve the reproducibility of the work that we publish. This form provides structure for consistency and transparency in reporting. For further information on Nature Portfolio policies, see our Editorial Policies and the Editorial Policy Checklist.

Statistics

For all statistical analyses, confirm that the following items are present in the figure legend, table legend, main text, or Methods section.

n/a	Confirmed	
☐	☒	The exact sample size (*n*) for each experimental group/condition, given as a discrete number and unit of measurement
☐	☒	A statement on whether measurements were taken from distinct samples or whether the same sample was measured repeatedly
☐	☒	The statistical test(s) used AND whether they are one- or two-sided<br>*Only common tests should be described solely by name; describe more complex techniques in the Methods section.*
☐	☒	A description of all covariates tested
☐	☒	A description of any assumptions or corrections, such as tests of normality and adjustment for multiple comparisons
☐	☒	A full description of the statistical parameters including central tendency (e.g. means) or other basic estimates (e.g. regression coefficient) AND variation (e.g. standard deviation) or associated estimates of uncertainty (e.g. confidence intervals)
☐	☒	For null hypothesis testing, the test statistic (e.g. *F*, *t*, *r*) with confidence intervals, effect sizes, degrees of freedom and *P* value noted<br>*Give P values as exact values whenever suitable.*
☒	☐	For Bayesian analysis, information on the choice of priors and Markov chain Monte Carlo settings
☒	☐	For hierarchical and complex designs, identification of the appropriate level for tests and full reporting of outcomes
☐	☒	Estimates of effect sizes (e.g. Cohen's *d*, Pearson's *r*), indicating how they were calculated

Our web collection on statistics for biologists contains articles on many of the points above.

Software and code

Policy information about availability of computer code

Data collection	No software was used for data collection.
Data analysis	The code used to analyse the data and generate the results can be found in the following repository: DOI: 10.5281/zenodo.8402455

For manuscripts utilizing custom algorithms or software that are central to the research but not yet described in published literature, software must be made available to editors and reviewers. We strongly encourage code deposition in a community repository (e.g. GitHub). See the Nature Portfolio guidelines for submitting code & software for further information.

Data

Policy information about availability of data

All manuscripts must include a data availability statement. This statement should provide the following information, where applicable:

- Accession codes, unique identifiers, or web links for publicly available datasets
- A description of any restrictions on data availability
- For clinical datasets or third party data, please ensure that the statement adheres to our policy

The data supporting the results can be found in: DOI: 10.5281/zenodo.8402455

Research involving human participants, their data, or biological material

Policy information about studies with human participants or human data. See also policy information about sex, gender (identity/presentation), and sexual orientation and race, ethnicity and racism.

Reporting on sex and gender	N/A
Reporting on race, ethnicity, or other socially relevant groupings	N/A
Population characteristics	N/A
Recruitment	N/A
Ethics oversight	N/A

Note that full information on the approval of the study protocol must also be provided in the manuscript.

Field-specific reporting

Please select the one below that is the best fit for your research. If you are not sure, read the appropriate sections before making your selection.

☐ Life sciences ☐ Behavioural & social sciences ☒ Ecological, evolutionary & environmental sciences

For a reference copy of the document with all sections, see nature.com/documents/nr-reporting-summary-flat.pdf

Ecological, evolutionary & environmental sciences study design

All studies must disclose on these points even when the disclosure is negative.

Study description	We use 10 well-resolved empirical bipartite networks from terrestrial habitats across the globe that contain information about both species distributions across multiple locations and their empirically-sampled biotic interactions (both mutualistic and antagonistic). Such newly available datasets allow us to directly compare, for the first time, co-occurrence and interaction networks, and we do so in terms of their degree distribution. We then examine how co-occurrence networks should be pruned to approximate features of the realised network of biotic interactions. We propose a simple model that uses knowledge not only on co-occurrence between species, but also on the frequency of such co-occurrence (that is, the number of sites where co-occurrence is known) to estimate the number of interactions of a species, and thus to predict the degree distribution of interaction networks based on information of species co-occurrence alone.
Research sample	The study uses 10 existing datasets involving different types of species interactions from different ecosystems and biomes, and spanning different spatial scales. All data used is already publicly available.
Sampling strategy	Each of the datasets used, followed different sampling strategies depending on the system analysed. Details about the sampling strategy of each dataset used are briefly explained in the methods section of this manuscript and appendix and explained in depth in the Nature Ecology and Evolution paper where the data was originally published (as explained in the text).
Data collection	The data collection was also specific of each dataset used.
Timing and spatial scale	The spatial scale differs across datasets but we take this into account given that we compare the network of co-occurrence with the network of interactions for each dataset.
Data exclusions	No data was excluded from the analyses.
Reproducibility	The study is not based on experimental findings. It is based on species interactions networks of empirical communities sampled in natural systems.
Randomization	We performed randomization to analyse the output of the proposed theoretical to prune the co-occurrence network.
Blinding	Blinding is not relevant in our study given that the data used describes species interaction networks based on empirical observations of natural systems and are not based on experimental communities in the lab.

Did the study involve field work? ☐ Yes ☒ No

Reporting for specific materials, systems and methods

We require information from authors about some types of materials, experimental systems and methods used in many studies. Here, indicate whether each material, system or method listed is relevant to your study. If you are not sure if a list item applies to your research, read the appropriate section before selecting a response.

Materials & experimental systems

n/a	Involved in the study
☒	☐ Antibodies
☒	☐ Eukaryotic cell lines
☒	☐ Palaeontology and archaeology
☒	☐ Animals and other organisms
☒	☐ Clinical data
☒	☐ Dual use research of concern
☒	☐ Plants

Methods

n/a	Involved in the study
☒	☐ ChIP-seq
☒	☐ Flow cytometry
☒	☐ MRI-based neuroimaging
